# The Influence of the Synthesis Method on the Characteristics of BaTiO_3_

**DOI:** 10.3390/ma16083031

**Published:** 2023-04-11

**Authors:** G. N. Almeida, R. N. de Souza, L. F. S. Lima, N. D. S. Mohallem, E. P. da Silva, A. M. A. Silva

**Affiliations:** 1 Mechatronic Engineering Department, Faculty of Technology, University of Brasília, Asa Norte, Brasília 70910-900, DF, Brazil; 2 Mechanical Engineering Department, Faculty of Technology, University of Brasília, Asa Norte, Brasília 70910-900, DF, Brazil; 3 Chemistry Department, Federal University of Minas Gerais, Av. Pres. Antônio Carlos, 6627, Pampulha, Belo Horizonte 31270-901, MG, Brazil

**Keywords:** BaTiO_3_ and titanate, sol-gel processes, electrical properties

## Abstract

In this work, barium titanate powders were produced by sol-gel and sol-precipitation methods from metal alkoxides. In the sol-gel method, tetraisopropyl orthotitanate was mixed with 2-propanol, acetic acid and barium acetate, and the gel samples obtained were calcined at 600 °C, 800 °C and 1000 °C. Through the sol-precipitation method, tetraisopropyl orthotitanate was mixed with acetic acid and deionized water and precipitated by the addition of a concentrated solution of KOH. The products were calcined at various temperatures, and the microstructural and dielectric properties of the BaTiO_3_ prepared for the two processes were analyzed and compared. The results of these analyses allowed us to observe an increase in the tetragonal phase and the dielectric constant (15–50 at 20 kHz) with increasing temperatures in the samples produced by the sol-gel method, while the sample obtained by sol precipitation was cubic. The presence of BaCO_3_ is more evident in the sample produced by sol-precipitation, and the band gap of the products obtained did not show significant variation, changing the synthesis method (3.363–3.594 eV).

## 1. Introduction

Recognized as one of the most important functional materials since 1964, barium titanate has been used in many practical applications in the form of single crystals, thin films and particulate materials [1]. It is an extremely important material for the electronics industry due to its high dielectric constant and spontaneous polarization, with a wide variety of industrial applications, such as multilayer ceramic capacitors (MLCs) and dynamic random-access memories (DRAMs). Particularly in the case of MLCs, nanoparticulate BaTiO_3_ with a high dielectric constant is necessary for the formation of thin dielectric layers [2].

**Figure 1:**
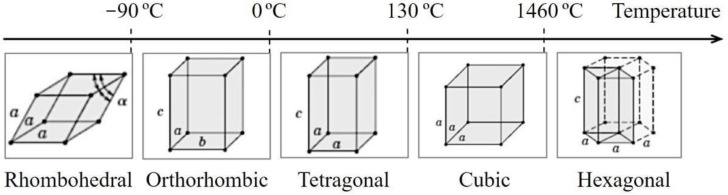
Phase transformations of the BaTiO_3_ crystal at different temperatures.

The crystalline structure and dielectric characteristics of BaTiO_3_ strongly depend on temperature applications. For crystals, when the temperature is below Curie’s temperature (~130 °C), the cubic structure is slightly distorted to a tetragonal ferroelectric structure with a dipole moment along the c [3] direction. Below 0 °C, the tetragonal structure turns into a ferroelectric phase. When the temperature is further reduced to −80 °C, it will transform to a rhombohedral structure. All phase transformations of the BaTiO_3_ crystal are illustrated in Figure 1.

Discussions about the dielectric properties of BaTiO_3_ crystals and ceramics should recognize that no “true” value can be cited. Almost all chemical and physical deviations from purity and perfection have a substantial effect on dielectric properties. For example, it was almost universally accepted that the Curie point of the pure crystal and conventional ceramic of BaTiO_3_ was 120 °C. Measurements on ultra-pure ceramics and crystals grown by the Remeika process [4] but without the addition of Fe^+3^ showed that their Curie point is close to 130 °C. The permittivity of the BaTiO_3_ ceramic depends strongly on the grain size. Pure BaTiO_3_ grain ceramics (a = 20–50 µm) show εr ≈ 1500–2000 at room temperature. At smaller grain sizes, there is a strong decrease in εr. However, in dense and fine-grained BaTiO3 ceramics, 1 µm-higher values (εr ≈ 3500–4000) can be observed [5].

The most common method of producing BaTiO_3_ has been the reaction between BaCO_3_ and TiO_2_ (anatase or rutile). For the reaction to occur, a homogeneous mixture and an accelerated diffusion reaction are required at temperatures ranging from 1000 °C to 1200 °C, resulting in particulates with an average size around or above 1 µm [6,7].

In the present work, we will discuss the synthesis of BaTiO_3_ nanometric powders from barium acetate and titanium (IV) isopropoxide as precursor materials. The products obtained through the sol-gel and sol-precipitation method were characterized by X-ray diffraction (XRD), scanning electron microscopy (SEM), UV-Vis spectroscopy and complex impedance. In this way, it was possible to calculate the crystallite size, cell parameters, tetragonality factor, band gap energy and dielectric constant of the produced samples.

## 2. Experimental Methods

### 2.1. Synthesis Sol Gel

**Figure 2:**
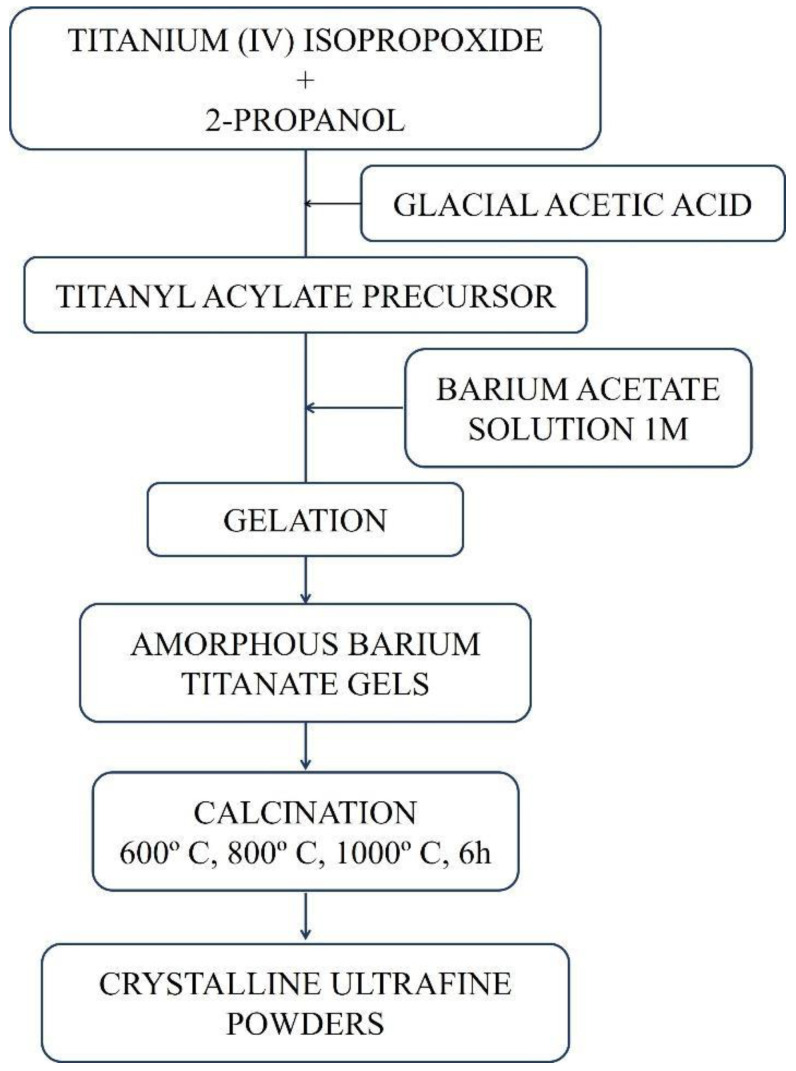
Diagram with the production stages of BaTiO_3_ by the sol-gel method.

BaTiO_3_ powders were synthesized by the sol-gel and sol-precipitation process. For the production of BaTiO_3_ through the sol-gel process (Figure 2), the titanium (IV) isopropoxide precursor, Ti(OC3H_7_)_4_ (Sigma Aldrich, 97%), was dissolved in 2-propanol (Sigma Aldrich, 99.5%) under constant stirring in a 1:10 molar ratio. Then acetic acid (Sigma Aldrich, 99.7%) was added until the solution reached pH = 3. Another solution containing barium acetate, Ba(CH_3_COO)_2_ (Sigma Aldrich, 99.7%), dissolved in deionized water (1 M) was prepared. The solution containing titanium alkoxide was added to the solution containing barium acetate under constant stirring in a 1:1 molar ratio. The obtained sol was gelled at room temperature. The gel samples obtained were calcined at 600 °C, 800 °C and 1000 °C for 6 h.

### 2.2. Synthesis Sol Precipitation

Tetraisopropyl orthotitanate and acetic acid were mixed in a 1:10 molar ratio. Then, deionized water was added until the formation of a white precipitate, which, after stirring, turned transparent. To this solution, a concentrated solution (pH > 13) of 5 M KOH was added, forming a white precipitate. The precipitate was reacted with a 1 M barium acetate solution in a Ba:Ti = 1:1 molar ratio at 100 °C with stirring and was kept under this temperature for 2 h. The powders were washed with deionized water to avoid potassium in the final composition and were subsequently dried in an oven at 110 °C. This synthesis procedure is very similar to the sol-gel synthesis of BaTiO3, but the alkaline conditions employed in this sol-precipitation process led to the formation of crystalline BaTiO_3_ powders without the heat treatment required in the sol-gel process [8,9].

**Figure 3:**
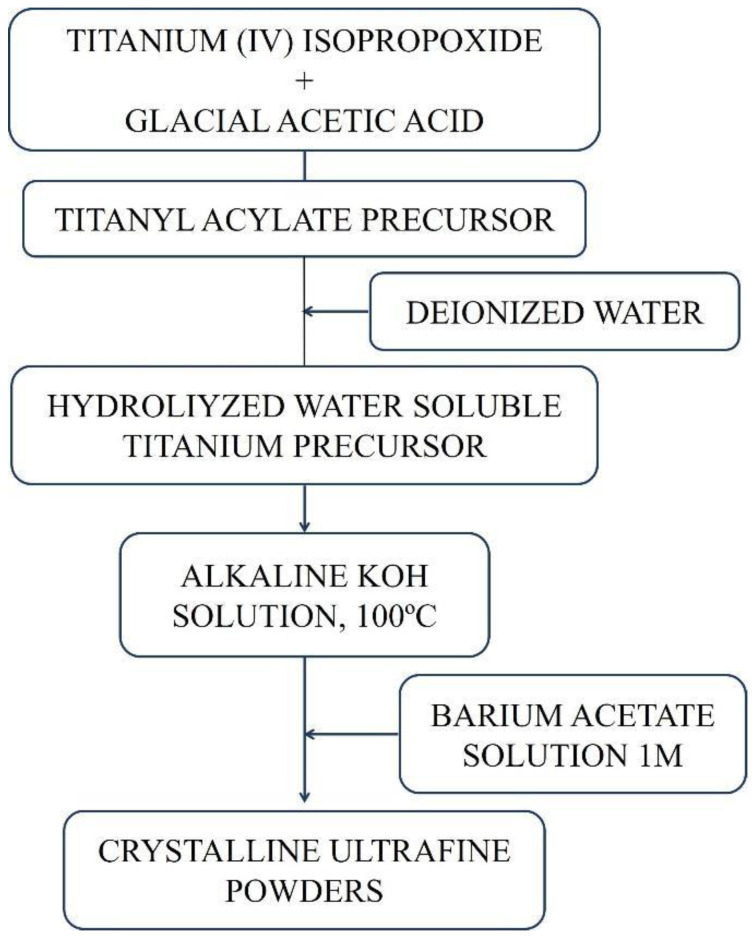
Diagram with the production stages of BaTiO_3_ by the sol-precipitation method.

The production stages of BaTiO_3_ by the sol-gel process and sol-precipitation method are summarized in Figure 2 and Figure 3, respectively.

### 2.3. Characterization

X-ray diffraction was used to quantify crystalline phases through their diffractometric patterns. The average size of the crystallite was determined by Scherrer’s equation:

(1) $$D_{hkl}=\frac{K\lambda }{\beta \cos\left(\theta \right)}$$

where:

*D*—Average particle diameter;

*K*—Constant that depends on the shape of the particles (sphere = 0.94);

*λ*—Wavelength of electromagnetic radiation;

*θ*—Diffraction angle;

*β* (2*θ*)—Width at half the height of the diffraction peak (FWHM).

The samples were analyzed in RIGAKU equipment, model ULTIMA IV, with Cu–Kα radiation and a Ni filter, under a voltage of 35 kV and a current of 15 mA. The diffractograms were obtained in the range of 2θ between 10° and 80° in the continuous mode, with a scanning speed of 0.5°/min and steps of 0.02°.

The micrographs were obtained using a Jeol-brand JSM-7100F scanning electron microscope (Brasília, Brazil) equipped with tungsten filament, with a resolution of 1.2 nm for a voltage of 30 kV and 3.0 nm at 1 kV, in order to qualitatively assess the degree of densification of the materials, as well as the average grain size.

The value of the band gap energies was determined using the Tauc/Davis-Mott model. To obtain information on the absorption of the samples, a UV-2450 spectrophotometer from Shimadzu (Brasília, Brazil was used, with a photometric precision of 0.3% T and a measuring range wavelength of 190~900 nm.

Impedance spectroscopy was used to observe the dielectric properties of the samples through an Agilent 4284A impedance meter, in a frequency range from f = 20 Hz to f = 1 MHz (f = ω/2π is the linear frequency). The powders were pressed uniaxially in a manual press, in pellets of approximately 12 mm in diameter and 4 mm in thickness. The samples in the cylindrical shape had their faces coated with a silver paste to perform the function of electrodes. All measurements were performed at room temperature.

## 3. Results and Discussion

**Figure 4:**
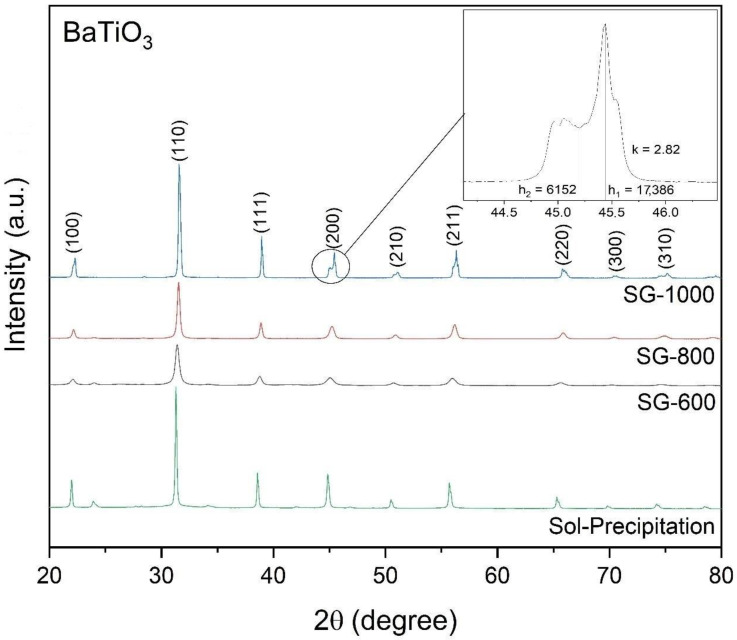
Diffraction pattern of the powders obtained by the sol-gel method, heat-treated at different temperatures (600 °C, 800 °C and 1000 °C) and synthesized via the sol-precipitation method. The detail shows the peak intensity for the plane (200) and the depression of the plane (002) of the sample calcined at 1000 °C.

The diffraction patterns used to identify the phases present in the samples, observed in Figure 4, reveal a cubic crystalline structure for the samples produced by the sol-gel method. However, the proximity between the network parameters a, b and c, observed in Table 1, for samples calcined at 600 °C and 800 °C (SG-600 and SG-800) and the low tetragonality index of these samples (1.0001 and 1.0011) are evidence of the crystalline phase. One of the characteristics of a cubic system is the exhibition of a single diffraction peak (002) at an angular position around 2θ = 45°. The unfolding of the originally cubic diffraction peak around 2θ = 45° occurs for the sample calcined at 1000 °C (detail of Figure 4) in (002) and (200), indicating the formation of the tetragonal phase. The k factor, defined as the ratio between the peak intensity for the plane (200), h_1_, and the intensity of the plane depression (002), h_2_ (h_1_/h_2_), of the tetragonal phase, indicates the relative ratio of the tetragonal phase to the non-tetragonal phase [10]. The detail in Figure 4 indicates the peak intensity for the plane (200), the depression of the plane (002) and the k factor of the sample calcined at 1000 °C, in which the diffraction peak unfolds around 2θ = 45°.

In Figure 4, it is also possible to observe the presence of some weak diffraction peaks, referring to barium carbonate, which can be found in positions 2θ ≅ 24°, 2θ ≅ 34° and 2θ ≅ 42°. The peaks appear discreetly at low temperatures, as in the diffractograms of the samples at 600 °C. As the heat treatment temperature increases, the peak intensity of BaCO_3_ decreases. At 1000 °C, the formation of the referred peaks is not observed. The increase in temperature suggests, as observed in the diffractograms of Figure 4, the decomposition of barium carbonate with the formation of barium titanate.

The presence of diffraction peaks referring to BaCO_3_ and a crystallite size slightly larger than those of the samples obtained by the sol-gel method is more evident for the sample obtained by the sol-precipitation method, as observed in Figure 4 and in Table 1. Another factor of great importance that differentiates the samples obtained by the two methods is the crystalline system. For obtaining a tetragonal structure by sol-gel, the material must be calcinated at 1000 °C; a lower temperature leads to a cubic phase in the same way as the sample produced by sol-precipitation, which is in accordance to what was reported by Yoon in 2006 [11].

**Figure 5:**
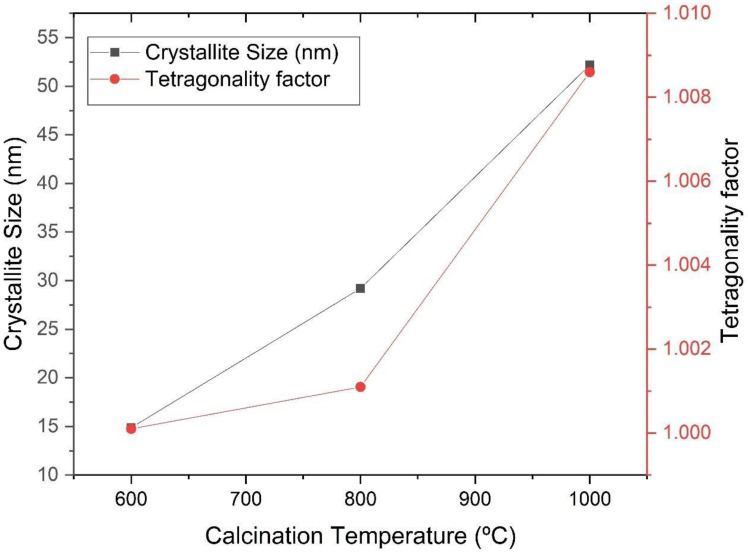
Size of the crystallite and tetragonality index as a function of the calcination temperature of the powders obtained by the sol-gel method and heat-treated at different temperatures (600 °C, 800 °C and 1000 °C).

**Figure 6:**
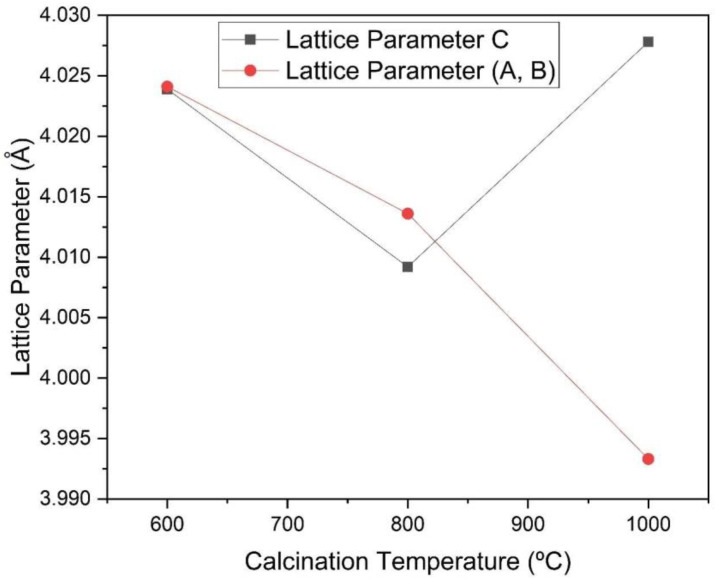
Network parameters as a function of the calcination temperature of the powders obtained by the sol-gel method and heat-treated at different temperatures (600 °C, 800 °C and 1000 °C).

The peaks identified in Figure 4 are characteristic of barium titanate. It can be seen in Figure 5 and Figure 6 that the increase in the calcination temperature causes an increase in the crystallite size, a reduction in the network parameters a and b and a consequent increase in the tetragonality factor, as the structure changes from cubic to tetragonal.

There is also an increase in crystallinity through the narrowing and increasing intensity of the diffraction peaks. The crystallite size has a strong influence on the tetragonality factor (c/a), on the crystalline structure and on the transition temperature of the cubic-tetragonal phase of BaTiO_3_ [12]. When non-aggregated powders are produced, crystallites do not find barriers to tetragonally distort. On the other hand, in the case of dense ceramics, the grain contours will hinder the tetragonal distortion of the structure. As the size of the crystallite increases, the tetragonality factor follows this trend, indicating a reduction in parameters a and b, as evidenced by the comparative analysis of the graphs in Figure 5 and Figure 6 [12]. The reduction in the network parameter c to 800 °C in Figure 6 suggests that there is an increase in the degree of aggregation of crystallites [12].

**Figure 7:**
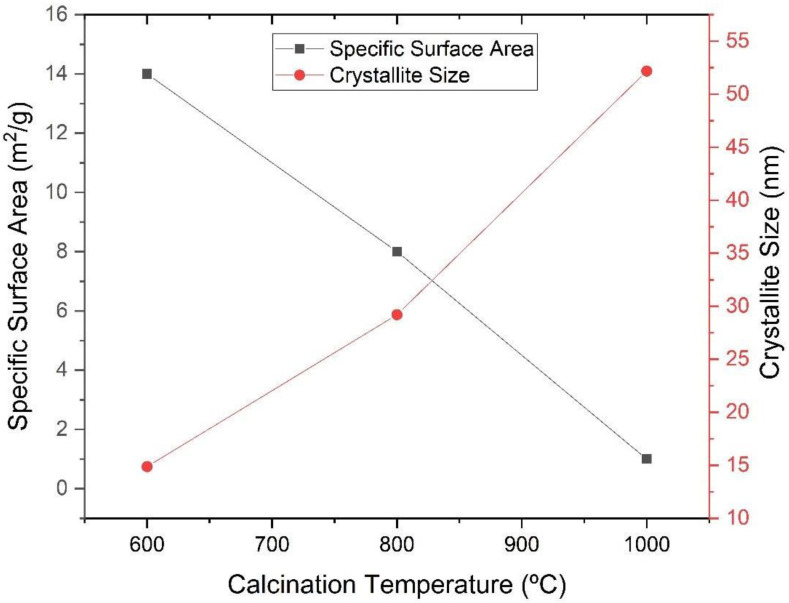
Specific surface area and crystallite size as a function of the calcination temperature of the powders obtained by the sol-gel method and heat-treated at different temperatures (600 °C, 800 °C and 1000 °C).

Figure 7 shows the evolution of the specific surface area and crystallite size with the increasing temperature of the calcination of the synthesized powder. A reduction in the specific surface area and an increase in the particle size are observed with the increase in the calcination temperature.

Considering that the particles of the synthesized materials have spherical or high-symmetry morphology and a small variation in particle size, the values of the specific surface area (*S_BET_*) and real density (*ρ*) can be used to calculate the average particle size using the following equation:

(2) $$D_{BET}=\frac{K}{\rho \cdot S_{BET}}$$

where *K* is a factor related to the particle shape. For isotropic and spherical particles, *K* = 6. Table 1 shows the average particle size (*D_BET_*) estimated from the specific surface area and theoretical density of BaTiO_3_, equal to 6.02 g/cm^3^.

**Figure 8:**
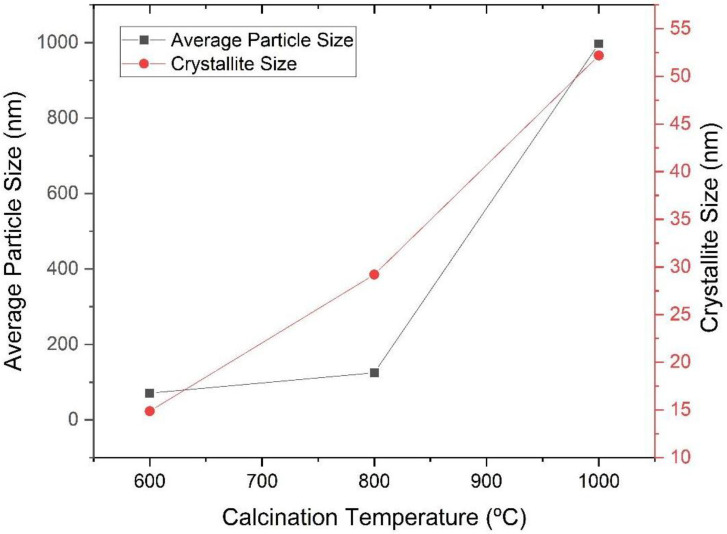
Average particle size (*D_BET_*) and crystallite size as a function of the calcination temperature of the powders obtained via sol-gel and heat-treated at different temperatures (600 °C, 800 °C and 1000 °C).

The data presented in Table 2 and Figure 8 reinforce the understanding of the tendency of the growth behavior of the particle with the increasing calcination temperature.

**Figure 9:**
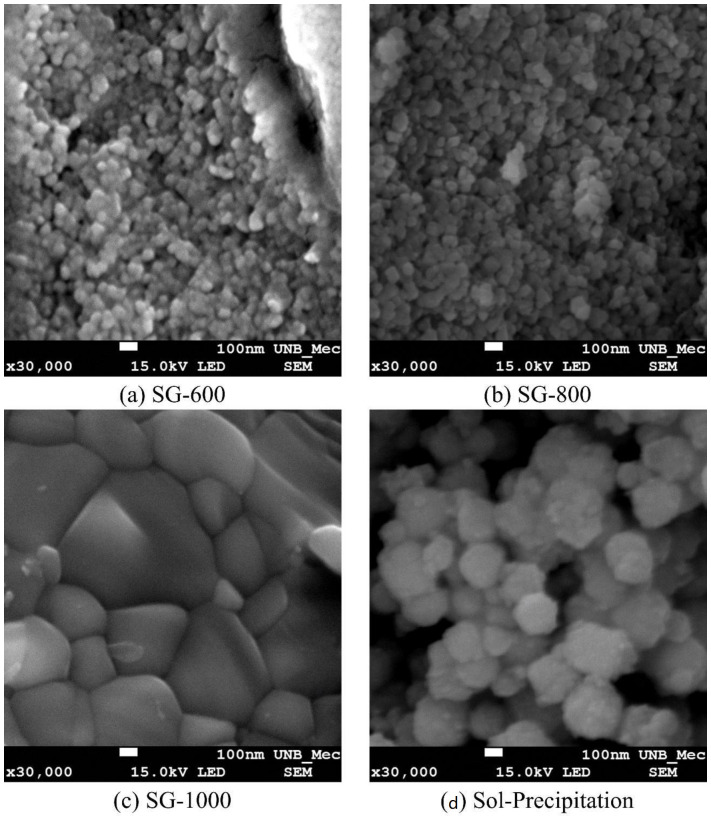
Scanning electron microscopy of powders synthesized by the sol-gel process at different temperatures: (**a**) 600 °C, (**b**) 800 °C, (**c**) 1000 °C and (**d**) sol-precipitation.

The micrographs (Figure 9) show that the powders obtained by the sol-gel method showed large clusters and/or aggregates, with a dense appearance and irregular sizes. In all samples, rods with sizes larger than the predominant particulates (BaTiO_3_) with rounded shapes were not observed. These rods are characteristic of BaCO_3_ [13].

The micrographs also show an increase in the grain size of the ceramics with the calcination temperature. From an analysis of the images, it is concluded that the powder obtained by the sol-gel process and calcined at 600 °C and 800 °C has an average size between 60 and 80 nm, respectively, while the calcined powder at 1000 °C consists of particles with an average size of 450 nm. The powders obtained by sol-precipitation had an estimated grain size of 220 nm.

**Figure 10:**
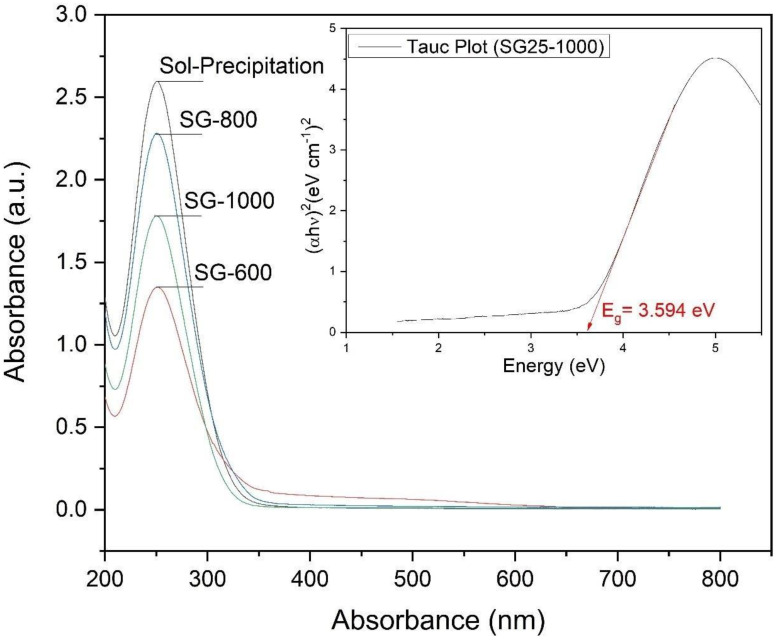
Graphics obtained from the UV-vis analysis of the powders synthesized by the sol-gel process at different temperatures (600 °C, 800 °C and 1000 °C) and by the sol-precipitation process.

The graphs obtained from the UV-vis analysis of the powders synthesized by the sol-gel process at different temperatures (600 °C, 800 °C and 1000 °C) and by the sol-precipitation process can be seen in Figure 10. The energy value of the forbidden band was estimated using the Wood–Tauc method.

**Figure 11:**
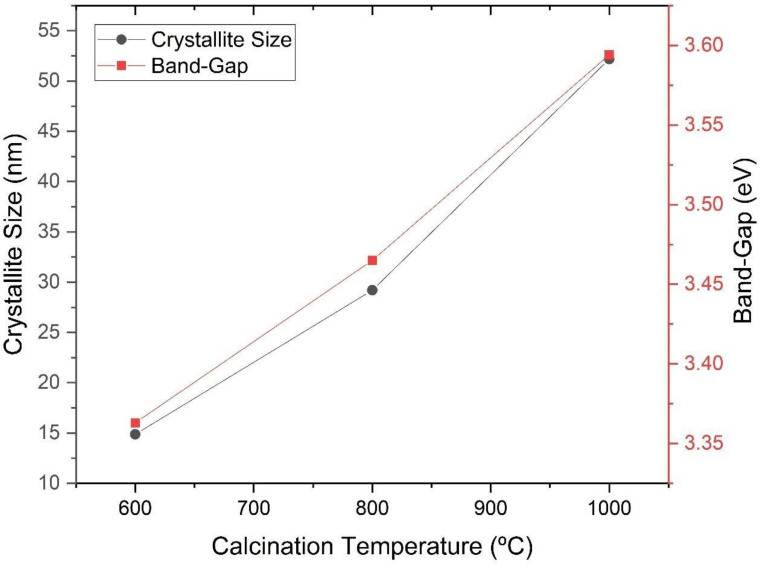
Band gap according to the size of the crystallites of the powders obtained by the sol-gel method and heat-treated at different temperatures (600 °C, 800 °C and 1000 °C).

Figure 10 suggests that all samples exhibit a spectrum with short bands, with a wavelength in the region close to the visible between 200 and 350 nm, with absorption peaks at 250 nm. The energy values of the forbidden gap, shown in Table 3, are very close for all samples, varying between 3.363 eV and 3.594 eV, suggesting that increasing the crystallite size increases the band gap as well, as can be seen in Figure 11. In an experimental study, the band gap of tetragonal barium titanate was reported to be around 3.40 V and is considered as an indirect and allowed band gap [14].

**Figure 12:**
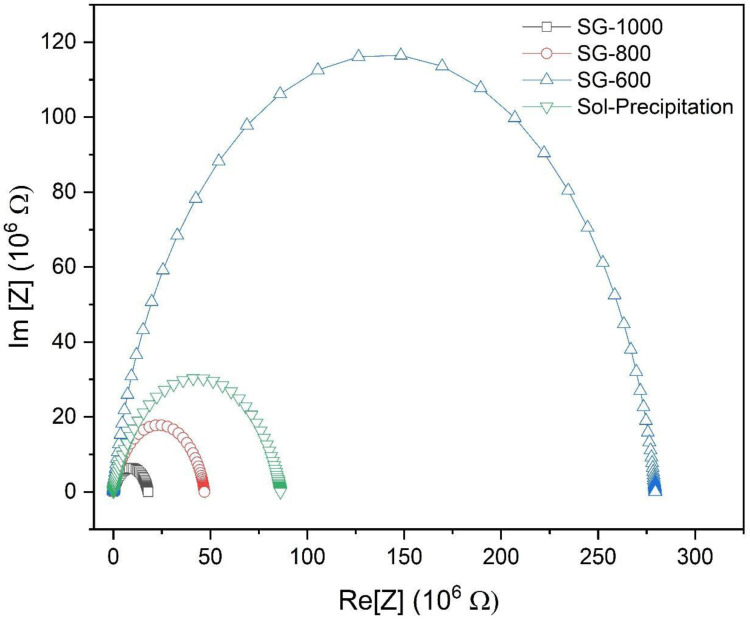
Complex impedance diagrams measured at room temperature, with an applied voltage of 1 V.

The impedance diagrams of the powders obtained by the sol-gel method and heat-treated at different temperatures (600 °C, 800 °C and 1000 °C) and sol-precipitation, measured at room temperature with an applied voltage of 1 V, can be seen in Figure 12. From an analysis of the diagram, it is possible to observe the effect of temperature on the material impedance.

The impedance spectrum is characterized by the appearance of semicircular arcs, whose pattern of evolution changes with increasing temperature. A variation in the perfect semicircular shape is due to the loss of energy at the interfaces and other defects present in the network [15]. The presence of two semicircular arcs that indicate the electrical processes related to the grain (a small arc usually formed at frequencies below 100 kHz) was not observed in the impedance diagram; only a single arc referring to the electrical processes related to the grain boundary was observed. It is possible to observe that the resistance of the grain is very small when compared to the resistance of the grain boundary contour, which corresponds to the semicircle diameter.

This result indicates that the calcination temperature was insufficient to generate a grain size for which the strength of the generated boundary was relevant [16].

**Figure 13:**
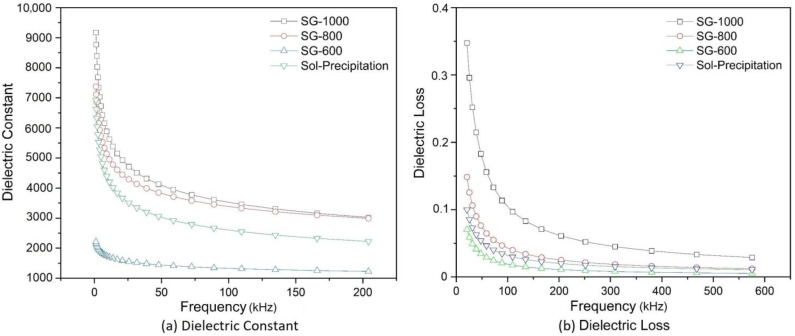
Variation in the (**a**) dielectric constant and (**b**) dielectric loss with the frequency at room temperature, with an applied voltage of 1 V.

Figure 13a shows the variation in the dielectric constant with the frequency for different calcination temperatures. The dielectric constant has higher values for calcined powders at higher temperatures and at lower frequencies (below 100 kHz); in fact, the dielectric constant is so high that the equipment could not accurately measure its constant below 20 kHz. For higher frequencies, little variation was observed for the electrical capacity values.

The grain size has a great influence on the dielectric properties of BaTiO_3_. At room temperature, grains of 0.8 ± 0.1 μm exhibit high values for the dielectric constant. However, for smaller grains, below 700 nm, the structure of the BaTiO_3_ ceramic changes from tetragonal to pseudo cubic, and the value of the dielectric constant tends to decrease. These results corroborate previous studies [5,17,18].

Figure 13b illustrates the dielectric loss of the powders obtained by the sol-gel method and heat-treated at different temperatures (600 °C, 800 °C and 1000 °C). The frequency range from 20 kHz to 575 kHz was used for the dielectric loss, and the range from 20 kHz to 210 kHz was used for the dielectric constant determination, those ranges were used since there was no dramatic change in both properties beyond those two upper frequencies. The graph shows a marked loss for frequencies below 100 kHz. The loss decreases, with the increasing frequency being lower for lower calcination temperatures. Table 4 summarizes the properties for each sample, such as the band gap energy, dielectric constant and loss, tetragonality factor and grain size.

In Table 4, it is possible to observe that samples with similar band gap values (such as SP and SG-800) have similar dielectric constants, and a higher band gap leads to a higher dielectric constant obtained. This tendency is in accordance with the literature and occurred linearly (adjusted equation: Y_(dielectric constant)_ = −949.19 + 291.52X_(band gap)_, with r^2^ = 0.9522) [19,20], corroborating the fact that the band structure of the material plays a more major role in the dielectric constants than the crystallite size itself.

## 4. Conclusions

In this work, it was possible to synthesize nanoparticles of BaTiO_3_ by the sol-gel and sol-precipitation methods. Both methods employed show excellent stoichiometric control, and it was possible to achieve a cubic phase by calcinating the sol-gel samples at 1000 °C.

The powders obtained by the sol-gel method showed large agglomerates and/or aggregates, with a dense appearance and irregular sizes, while the powder obtained by the sol-precipitation method has a less dense appearance and regular sizes. The diffraction peaks referring to barium carbonate appear more significantly in the sample produced by sol-precipitation, as it was not calcinated at higher temperatures for it to be eliminated. In samples produced by the sol-gel method and calcinated at 800 and 1000 °C, these peaks are absent. Despite the small variation in the band gap of all the samples analyzed, there is a clear relationship between this property and the size of the crystallite obtained. In that sense, it was possible to infer that both the crystallite size and the band gap of samples can be used for tailoring the dielectric constant of barium titanate, At last, it was possible to observe, by complex impedance, that high temperatures are favorable for dielectric polarization, because the dielectric constant increased with temperature, and this property also showed a dependance on the synthesis method, crystalline phase and band gap of the material, achieving higher results for the tetragonal samples calcinated at 1000 °C.

## Figures and Tables

**Table 1:** Average size of crystallites obtained by the Scherrer equation and tetragonality index of the powders obtained by the sol-gel method and heat-treated at different temperatures (600 °C, 800 °C and 1000 °C) and by the sol-precipitation method.

Sample	D (nm)	a, b (Å)	c (Å)	FWHM (2θ ≈ 31°)	Crystalline System
SG-600	14.88	4.0241	4.0239	0.42°	Tetragonal ICSD 86286
SG-800	29.21	4.0136	4.0092	0.26°	Tetragonal ICSD 73642
SG-1000	52.18	3.9933	4.0278	0.16°	Tetragonal ICSD 28620
Sol-Precipitation	59.72	4.0386	4.0386	0.13°	Cubic ICSD 28851

**Table 2:** Values related to the specific area (*S_BET_*) and average particle size *(D_BET_*) of the powders obtained by the sol-gel method and heat-treated at different temperatures (600 °C, 800 °C and 1000 °C) and by the sol-precipitation method.

Sample	Specific Area (m^2^/g)	Average Particle Size (nm)
SG-600	14	71
SG-800	8	125
SG-1000	1	997
SP	31	32

**Table 3:** Values found by the Tauc method for the band gap of samples obtained by the sol-gel and sol-precipitation method.

Sample	Band Gap (eV)
SG-600	3.363
SG-800	3.465
SG-1000	3.594
Sol-Precipitation	3.528

**Table 4:** Table comparing samples with the respective band gap energy, dielectric properties, tetragonality factor and grain size at room temperature.

Sample	Band Gap (eV)	Dielectric Constant @ 50 kHz	Dielectric Loss @ 50 kHz (%)	Resistance (MΩ)	Tetragonality Factor (-)	Crystallite Size (nm)
SG-600	3.363	2400	8	278	1.0001	60
SG-800	3.465	7400	15	48	1.0011	80
SG-1000	3.594	9300	34	23	1.0090	450
SP	3.528	7900	10	85	1.0000	220

## Data Availability

Not applicable.
